# Signatures of transient Wannier-Stark localization in bulk gallium arsenide

**DOI:** 10.1038/s41467-018-05229-x

**Published:** 2018-07-23

**Authors:** C. Schmidt, J. Bühler, A.-C. Heinrich, J. Allerbeck, R. Podzimski, D. Berghoff, T. Meier, W. G. Schmidt, C. Reichl, W. Wegscheider, D. Brida, A. Leitenstorfer

**Affiliations:** 1 Department of Physics and Center for Applied Photonics, University of Konstanz, D-78457 Konstanz, Germany; 2 Department of Physics and Center for Optoelectronics and Photonics Paderborn, University of Paderborn, D-33098 Paderborn, Germany; 3 Solid State Physics Laboratory, ETH Zurich, CH-8093 Zürich, Switzerland

## Abstract

Many properties of solids result from the fact that in a periodic crystal structure, electronic wave functions are delocalized over many lattice sites. Electrons should become increasingly localized when a strong electric field is applied. So far, this Wannier–Stark regime has been reached only in artificial superlattices. Here we show that extremely transient bias over the few-femtosecond period of phase-stable mid-infrared pulses may localize electrons even in a bulk semiconductor like GaAs. The complicated band structure of a three-dimensional crystal leads to a strong blurring of field-dependent steps in the Wannier–Stark ladder. Only the central step emerges strongly in interband electro-absorption because its energetic position is dictated by the electronic structure at an atomic level and therefore insensitive to the external bias. In this way, we demonstrate an extreme state of matter with potential applications due to e.g., its giant optical non-linearity or extremely high chemical reactivity.

## Abstract (web summary)

In strong enough electric fields the non-linear response of electrons in crystals is expected to lead to spatial localization but so far this has only been seen in artificial structures. Schmidt et al. present evidence of this Wannier-Stark localization effect in bulk GaAs driven by intense mid-infrared pulses.

## Introduction

Strong optical biasing of solid-state materials with few-cycle laser pulses can trigger extremely non-linear polarization responses^1^. Analysis of these non-perturbative excitations has provided information on fundamental properties such as electronic band structures^2,3^ and on ultrafast phenomena like dynamical Bloch oscillations^4^, interference between crystal electrons^5^ or attosecond transmission changes due to Franz–Keldysh effects^6^. Theoretical understanding of such prototypical solid-state phenomena started emerging in 1929, when Felix Bloch laid foundations for a quantum description of electrons in a periodic potential^7^. A few years later, nonlinear phenomena that might occur at high electric fields were first discussed explicitly by Clarence Zener^8^. Subsequently, a series of papers by Gregory H. Wannier pointed out the relevance of localized wave functions for understanding the charge dynamics in periodic potentials superimposed with macroscopic fields^9^. It was predicted that in extremely high fields, even delocalized electronic states might become confined to the atomic scale^7–9^, establishing the regime of Wannier–Stark localization. It took half a century for experimental evidence to emerge of the spectral Wannier–Stark ladder^10,11^ and its time-domain counterpart, Bloch oscillations of band electrons^12,13^. Both phenomena were demonstrated in semiconductor superlattices^14^, where a one-dimensional periodic superpotential for electrons is established as a consequence of alternating layers of materials with different bandgap energy. So far, those conditions have been deemed out of reach in three-dimensional solid-state crystals without mesoscopic superstructure because of the high electric field *E*_WSL_, where Wannier–Stark localization should set in^10^:

1 $$\begin{array}{c} {E_{{\mathrm{WSL}}} \approx \frac{{ {\Delta }}}{{eD}}} \end{array}$$

with the energetic width of electronic energy bands *Δ*, the elementary charge *e* and the spatial displacement *D* under which the periodic potential is invariant. In a GaAs/AlGaAs superlattice with a period *D* around 50 Å, the width *Δ* of the first electronic miniband is of the order 30 meV and therefore, *E*_WSL_ amounts to approximately 60 kV/cm. Such an electric field is easily sustained by typical semiconductor devices. The situation changes drastically when considering bulk solids, where *D* is in the order of the lattice constant *a* ≈5 Å and *Δ* ≈1 eV, resulting in *E*_WSL_ ≈20 MV/cm, which is far above the typical breakdown field of dielectrics under stationary bias. In contrast to superlattices where localization concerns only the envelope wave functions of electrons in the modulated compound structure, such Wannier–Stark quantization in a bulk material would result in strong distortions of the electronic system on the level of a single unit cell, equivalent e.g., to a significant change of interatomic chemical bonds.

In this work, we access these extreme physical conditions by transiently biasing bulk GaAs with phase-stable optical field transients. As a result, we observe a sudden blueshift of the absorption edge of the semiconductor which we interpret as a signature of the transition of electronic system from three- to two-dimensional character.

## Results and discussion

### Experimental principle and electronic structure of GaAs

**Fig. 1:**
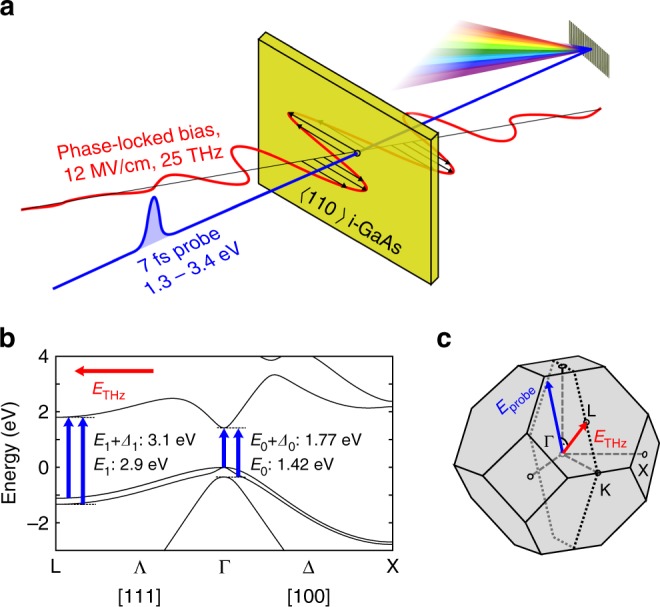
Experimental scheme and fundamental materials properties of GaAs. **a** A few-cycle mid-infrared waveform centered at 25 THz (sketched in red) propagates into a <110>-oriented thin film of intrinsic GaAs (yellow). The electric field amplitude reaches up to 12 MV/cm inside the material, with polarization set to the [111] crystallographic direction. The resulting changes in interband electronic absorption are probed at variable times relative to the biasing field, with weak 7-fs optical pulses containing photon energies from 1.3 eV to 3.4 eV (blue). After transmission through the sample, the broadband probe pulses are spectrally dispersed with a monochromator and detected with a CCD array. **b** Electronic band structure of GaAs at room temperature, with interband critical points and transition energies relevant to the experiment depicted by blue arrows. The direction of the mid-infrared biasing field *E*_THz_ is indicated in red. **c** First Brillouin zone of the zincblende-type crystal structure of GaAs, with direction of biasing field **E**_THz_ and probe polarization **E**_probe_ given by red and blue arrows, respectively. The dotted line indicates the intersection with the <110>-oriented surface of samples

A schematic sketch of the experimental principle is depicted in Fig. 1a. The mid-infrared (MIR) center frequency of biasing transients (red line) of 25 THz is far below the interband resonance at 350 THz that corresponds to room-temperature fundamental bandgap energy of *E*_0_ = 1.424 eV, ensuring an adiabatic (i.e., non-dissipative and quasi-instantaneous) response of the electronic system. On the other side, the spectral content of our few-cycle bias field does not overlap with optical phonon frequencies (*ν*_TO_ = 8 THz and *ν*_LO_ = 8.8 THz in GaAs), resulting in negligible motion of the ion lattice. We probe the interband electronic absorption with near-infrared and visible laser pulses of a duration of 7 fs, which is significantly shorter than the half-cycle period of our bias field of 20 fs (indicated as a blue line in Fig. 1a). After transmission through a <110>-oriented thin film of single crystal and intrinsic GaAs (depicted yellow in Fig. 1a), the change in transmission is recorded as a function of the relative arrival time with respect to the biasing waveform and spectral position within the broadband probe pulses. Detailed information on the experimental setup and samples is provided in Methods and Supplementary Fig. 1. The electric field vector of the MIR bias is oriented parallel to the [111] crystallographic axis, i.e., along the body diagonal of the cubic unit cell of GaAs (Supplementary Fig. 2a). In this Λ-direction towards the L-point (see Fig. 1c), the energetic width of both valence and conduction bands is minimal with *Δ* ≈ 1 eV (Fig. 1b). According to Eq. (1), also the lattice period *D* enters into the expression for the electric field where Wannier–Stark localization sets in. In contrast to a one-dimensional situation, it is not a priori clear which length determines *D* in three dimensions: in the [111] direction, the distance between lattice planes is maximized to *a*/$\sqrt 3$ = 3.2 Å and the first Brillouin zone exhibits its minimum extent of $\pi \sqrt 3$/*a*. However, the entire structure maps onto itself only after translation by a full-body diagonal of the cube equaling $\sqrt 3$*a* = 9.8 Å. These two options are opening up a potential range for *E*_WSL_ between 10 MV/cm and 30 MV/cm. Since GaAs lacks inversion symmetry, transmission changes linear in the electric field might emerge, which are sign dependent and not included in our discussion. However, such electro-optic effects cancel out by symmetry when the probe polarization is set perpendicular to a bias in [111] direction (see Supplementary Fig. 2).

### Moderate mid-infrared bias fields

**Fig. 2:**
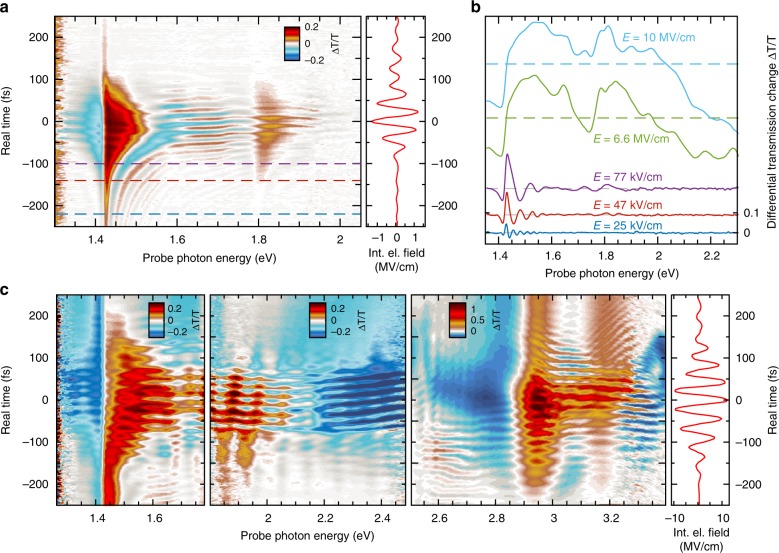
Subcycle electroabsorption of intrinsic GaAs. **a** Moderate field level. Left: Differential transmission changes, Δ*T*/*T*, color-coded as a function of probe photon energy and time; right: electric field (red) of biasing mid-infrared transient with maximum amplitude of 1.4 MV/cm as a function of time. **b** Temporal slices of Δ*T*/*T* as a function of probe photon energy, as taken along different contours with constant electric field amplitudes *E*. **c** Intense electric field level. Left three panels: Δ*T*/*T* color-coded as a function of probe photon energy and time, as measured with three different probe spectra depicted in Supplementary Fig. 1b. Data above 2.5 eV were obtained with a GaAs epitaxial film of 100 nm thickness, while all other spectra were measured on a 400-nm-thick sample; right: electric field trace (red) of biasing transient with maximum amplitude of 12 MV/cm

In Fig. 2a, differential transmission changes, Δ*T*/*T,* induced in a GaAs sample of 400 nm thickness are color-coded as a function of time (vertical axis) with respect to the maximum of the single-cycle biasing field transient of 1.4 MV/cm (red graph at right) and probe photon energy (horizontal axis). Two distinct spectral regions are immediately apparent in this regime of moderate electric fields: in the time interval between *t* = −100 fs and +100 fs, pronounced signatures of transient absorption (blue, Δ*T*/*T* < 0) and induced transmission (red, Δ*T*/*T* > 0) are separated by a probe photon energy of *E*_pr_ = 1.42 eV close to the fundamental bandgap *E*_0_. These features have been attributed to the dynamical Franz–Keldysh effect in semiconductors under multicycle mid-infrared biasing with comparable peak fields^15,16^. Owing to our broadband probe, we can see a similar, but weaker pattern repeated around an energy of *E*_0_ + *Δ*_0_ = 1.77 eV, which corresponds to transitions from the split-off valence band into the Γ-minimum of the conduction band (see Fig. 1b). In addition, pronounced regions of alternating induced absorption and bleaching appear above the fundamental gap before *t* = −100 fs. At those early times, the electric field is much smaller than the maximum of the biasing transient, and spectral oscillations are arranged in hyperbola-like contours. The physical origin of these signatures becomes clear looking at four slices in the lower part of Fig. 2b, which were taken at the time horizons indicated by dashed lines with identical color coding in Fig. 2a: the transmission changes correspond to the spectral oscillations expected from Franz–Keldysh electro-absorption within the interband continuum^17,18^. Interestingly, these features are not modulated in the time domain by biasing optical cycles. At low fields, interband dephasing close to the bandgap is considerably longer than a half-cycle period of the biasing transient. The precise arrival time of the probe pulse exciting the interband polarization is irrelevant in this regime because differential electro-absorption is averaged over the dephasing time. Only for times above *t* = −100 fs does the bias field become strong enough to shorten the interband dephasing to a value comparable to the half-cycle period of 20 fs. Under these conditions, a clear temporal modulation of differential absorption manifests itself in horizontally oriented differential transmission patterns with twice the periodicity of the biasing transients that are visible in Fig. 2a.

### Exploratory theoretical modeling with cosine-like bands

**Fig. 3:**
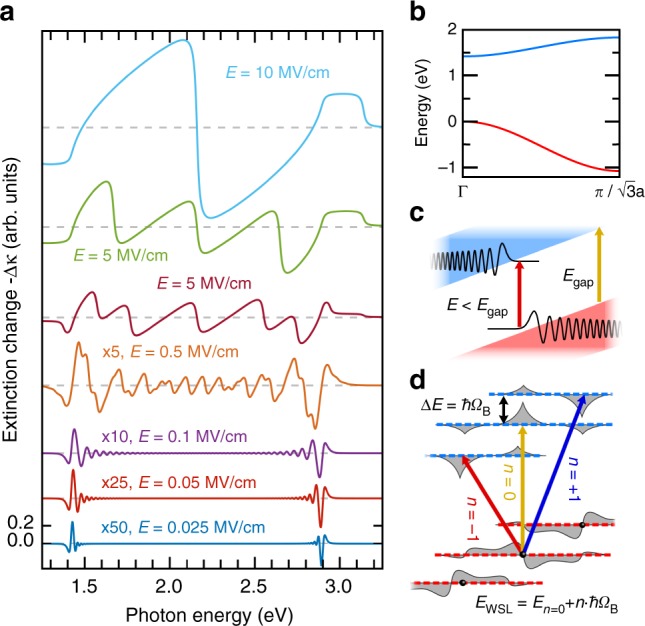
Simulations with cosine-like bands and electro-absorption regimes. **a** Negative extinction change, −Δκ, with respect to the unbiased case versus photon energy and for various stationary electric fields *E*, as calculated for the cosine-like two-band model depicted in **b**. **c** Sketch of conditions for Franz–Keldysh effect (*E* ≤ 0.5 MV/cm in **a**). Airy-type envelope wave functions extending over many lattice sites lead to finite transition probabilities for energies below the unbiased bandgap energy *E*_gap_ (red arrow) and oscillating absorption changes in the interband continuum above *E*_gap_ (orange arrow). Valence and conduction bands are indicated in red and blue, respectively. **d** Visualization of the origin for the Wannier–Stark ladder (*E* > 0.5 MV/cm in **a**). In the high-field regime, wave functions (gray) are confined to a small number of lattice sites. This leads to discrete transition energies for electrons within the same site (orange arrow, *n* = 0) and between different sites (red and blue arrows, *n* = ± 1) which are separated by the Bloch energy Δ*E* = *ħΩ*_B_. *n* is the spatial index of a step in the Wannier–Stark ladder

To understand the physical mechanisms of electro-absorption for extremely high electric fields of this study, we have carried out theoretical calculations based on increasingly realistic model assumptions (see Methods). In Fig. 3a, the extinction changes, −Δκ, for various static electric fields, *E*, are plotted as a function of probe photon energy for a model assuming one purely cosine-like valence and conduction band, respectively (see Fig. 3b). The energetic width of both bands is adjusted to match the total bandwidth of interband absorption in [111] direction (compare Fig. 1b), and *D* is set to $\sqrt 3$*a*. For *E* *<* 100 kV/cm, Franz–Keldysh signatures similar to the ones found experimentally in Fig. 2b are calculated at the *E*_0_ fundamental bandgap, around 1.42 eV. Microscopically, these spectral oscillations are due to the airy-like character of the electronic wave functions in such moderate fields^17,18^, which are modulated on a length scale much longer as compared to the unit cell of the crystal (see Fig. 3c). In this limit, electro-absorption remains perturbative and may be described within an effective mass approximation. As expected for the highly symmetric band dispersion adopted here, analogous features appear also at the upper edge of the electronic absorption band around 2.9 eV, but they are inverted in amplitude due to the opposite sign of the interband-reduced mass at the L-point (see Fig. 3b). The transition to non-perturbative conditions occurs when Franz–Keldysh signatures from the upper and lower absorption edges become so extended in photon energy that they meet in the center of the interband transition region^19^. In Fig. 3a, this regime starts at *E* = 0.5 MV/cm with the presence of sharp steps occurring in Δκ. They correspond to the Wannier–Stark ladder that emerges due to the localization of electronic wave functions within a small number of lattice sites, as sketched in Fig. 3d. The central edge at *E*_pr_ = 2.15 eV belongs to spatially direct transitions (green arrow in Fig. 3d) between states peaked within the same unit cell. Additional edges are spaced by Δ*E* = *ħΩ*_B_, starting from the central one where

2 $$\begin{array}{c} {{ {\Omega }}_{\mathrm{B}} = \frac{{eED}}{\hbar }} \end{array}$$

is the famous Bloch frequency^8,9^. They are due to spatially indirect transitions between electronic states centered at different lattice sites, as indicated by red and blue arrows in Fig. 3d. At an electric field *E* = 10 MV/cm, the steps in the Wannier–Stark ladder become wider than the width of the interband transition region, and only the central edge remains (see Fig. 3a).

### Wannier–Stark regime at high mid-infrared bias fields

In order to search for manifestations of such phenomena in the experiment, we now increase the peak electric field of the biasing transient to 12 MV/cm (see red graph in the rightmost panel in Fig. 2c) and record the interband transmission changes in the interval of probe photon energies from 1.25 eV to 3.4 eV. This wide range of *E*_pr_ is covered by using the red, green and blue probe spectra depicted in Supplementary Fig. 1b, corresponding to three separate two-dimensional data panels starting at the left side of Fig. 2c, respectively. Note that Δ*T*/*T* between 2.5 eV and 3.4 eV was measured in a GaAs sample as thin as 100 nm because of the extremely short absorption length in this range. The salient features found in Fig. 2c are as follows: at the *E*_0_ fundamental gap of 1.42 eV, induced absorption (blue) and bleaching (red) still occur analogous to the moderate-field scenario presented in Fig. 2a, and even the hyperbola-like contours related to Franz–Keldysh oscillations arise at very early delay times. However, a completely new feature turns up around *E*_pr_ = 2.1 eV, consisting of an abrupt change from induced transmission (red) to transient absorption (blue). This step in Δ*T*/*T* occurs for every half-cycle in the entire central region of the biasing transients between *t* = −100 fs and +100 fs, where absolute field levels exceed approximately 6 MV/cm. In this interval, the transient differential transmission changes remain negative (blue) all the way up to *E*_pr_ = 2.9 eV. From 2.9 eV to 3.2 eV, two distinct features of subcycle-induced transmission (red) are found in the high-field temporal region which can be associated to a bleaching of *E*_1_ and *E*_1_ + *Δ*_1_ critical point transitions along the Λ-direction in the band structure (see Fig. 1b). This entire scenario is strikingly similar to our simulation result at *E* = 10 MV/cm in Fig. 3a, which also predicts an edge in differential transmission at *E*_pr_ = 2.15 eV.

### Realistic bands and transient biasing

**Fig. 4:**
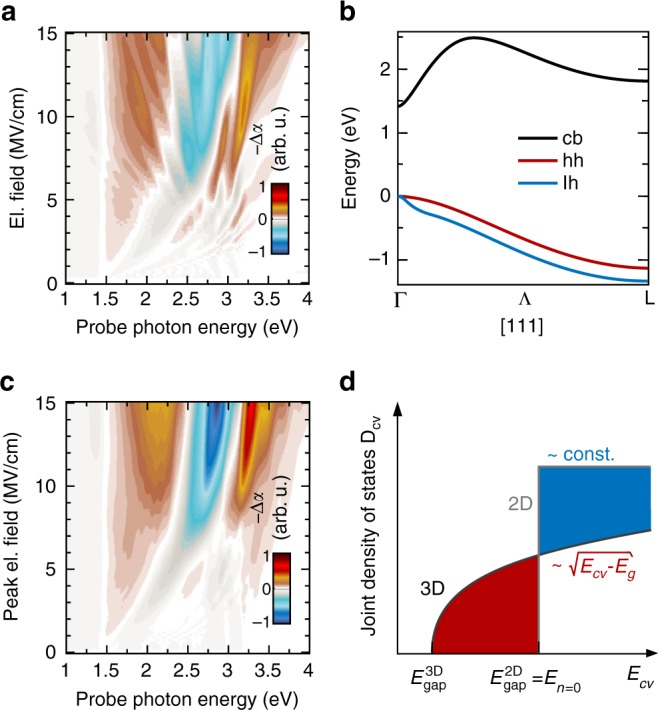
Simulations with realistic band dispersions. **a** Negative absorption change −Δα with respect to the unbiased case color-coded versus photon energy and stationary electric fields, as obtained adopting the GaAs light-hole (lh, blue), heavy-hole (hh, red) and conduction band (cb, black) dispersions along the Λ-direction in **b**. Details are explained in Methods. **c** Negative absorption change -Δα color-coded versus photon energy, as expected when probing in the field maximum of an oscillatory bias of 25 THz frequency. **d** Visualization of the change in joint density of states *D*_*cv*_ as a function of relative energy *E*_*cv*_ between valence and conduction bands. Omitting excitonic effects, *D*_*cv*_(*E*_*cv*_) is proportional to the absorption constant α_*cv*_(*E*_*cv*_). In case of a three-dimensional semiconductor with parabolic bands, *D*_*cv*_ increases square-root-like starting at the bandgap energy *E*_gap_^3D^. As wave functions become localized in one direction, *D*_cv_ changes to the step-function-like behavior characteristic for two-dimensional electronic systems. A larger bandgap energy *E*_gap_^2D^ results, corresponding to the transition energy *E*_*n*=0_ of the central step in the Wannier–Stark ladder (see Fig. 3d). When measuring the differential transmission change between both scenarios, a characteristic bleaching region (shaded in red) occurs below and induces absorption (blue) above *E*_gap_^2D^

We now discuss the important differences between the experiment and the model results of Fig. 3. First, there are no pronounced Franz–Keldysh-like spectral oscillations in measurements around the *E*_1_ and *E*_1_ + *Δ*_1_ critical point transitions even at low MIR bias fields. Second and most strikingly, we were unable to detect clear evidence of additional steps at field-dependent positions in the interband absorption that might be due to the spatially indirect transitions in the Wannier–Stark ladder (see Fig. 3a, d). To understand these facts, we first turn towards a more accurate description of the GaAs band structure. In Fig. 4a, the absorption change under stationary electric bias is color-coded versus probe photon energy adopting realistic dispersions in [111] direction for conduction, heavy-hole and light-hole bands (see Fig. 4b and Methods). The presence of two valence bands together with non-monotonous characteristics of the conduction band produces more structured and asymmetric electro-absorption features, as compared to the simplified model in Fig. 3. Microscopically, this finding is due to the following effect^20^: the non-cosine-like shape of electronic bands in realistic three-dimensional solids arises from overlap of atomic wave functions beyond the nearest neighboring lattice sites. In this case, field dependence of indirect features in Wannier–Stark electro-absorption becomes rather complex with a tendency to average out. Therefore, the central step in the Wannier–Stark ladder becomes dominant because its energetic position is independent of the electric field to first order. These facts are studied in detail by the discussion around Fig. 3 of ref.^20^. Still, some field-dependent substructures due to spatially indirect transitions in the Wannier–Stark ladder remain discernible. To also take into account the dynamical aspects in our experiment, the absorption changes expected when probing around the field maxima of an external bias oscillating with a frequency of 25 THz are modeled in Fig. 4c including an interband dephasing time of 20 fs. In this case, an abrupt change between bleaching (red) and induced absorption (blue) sets in around a probe photon energy of 2.2 eV at a peak field of approximately 6 MV/cm and slightly blueshifts when the bias is increased up to 15 MV/cm. This simulation agrees well with the experimental high-field data in Fig. 2b, c. The step-like character and field-independent position of the central feature in Wannier–Stark electro-absorption is explained microscopically in Fig. 4d; without external field, we are dealing with a three-dimensional direct-gap semiconductor which exhibits a square-root-like behavior of the joint density of states above the fundamental absorption edge, *E*_gap_^3D^ = *E*_0_. When an electric field of the order of *E*_WSL_ is applied, localization of wave functions occurs only in one lattice direction, while the electronic bonding structure perpendicular to the field remains intact. A step-function-like absorption spectrum characteristic for two-dimensional electron systems arises with an onset of absorption at a higher energy *E*_gap_^2D^, which may be related to the atomic transition energies in the compound (Fig. 3d). The differential transmission change, Δ*T*/*T,* mirrors the difference between the two scenarios, as indicated by the red- and blue-shaded areas in Fig. 4d. When taking a spectral slice along a contour line induced by a half-cycle with 6.6 MV/cm peak electric field (see Fig. 2b and Methods), we do find the onset of this effect. Moving along a contour corresponding to 10 MV/cm, a clear step at *E*_pr_ = 2.1 eV arises in Fig. 2b owing to the transfer of spectral weight illustrated by Fig. 4d. Consequently, we regard this fact as direct evidence for Wannier–Stark localization.

### Possible signatures of isolated atomic transitions

Interestingly, the abrupt shift of the absorption edge from *E*_0_ = 1.42 eV to 2.1 eV occurring at ultrahigh electric bias fields might indeed relate to genuine electronic transitions of isolated constituent atoms^21^: the ^2^P^o^_0½_ state of the As atom is located 2.25 eV above the ^4^S^o^_1½_ ground state. First-principles local density calculations^22^ of the electronic structure of GaAs show that the topmost valence bands are composed almost exclusively of 4p orbitals of the As atoms, while the first conduction band exhibits strong contributions from both As and Ga. Consequently, some net transfer of electronic charge from As to Ga upon absorption of a photon may be required to establish the minimum transition energy at the fundamental *E*_0_ gap. With the onset of Wannier–Stark quantization, wave functions become more localized, photoexcited valence electrons should remain more readily at the As sites and therefore, the absorption edge might move close to a bare atomic transition of this species. Also note that in turn, the energies of *E*_1_ and *E*_1_ + *Δ*_1_ critical points (Fig. 1b) which strongly bleach in high electric fields (Fig. 2c) are located close to the 3.074-eV transition from the 4*p* ^2^P^o^_0½_ ground state to the 5*s* ^2^S_0½_ level of the isolated Ga atom^21^.

### Influence of interband tunneling

Owing to the ultrashort character of our few-cycle and mid-infrared biasing pulses, transmission changes from free carriers generated via interband tunneling^23^ are negligible during the time when high external fields are applied to our samples. Influences from this process together with subsequent avalanche multiplication become relevant only at delay times beyond ~500 fs. A detailed investigation of this context is provided by the Methods section and Supplementary Figs. 3 to 5. We also want to emphasize that high harmonics of our biasing central frequency of 25 THz are emitted under conditions equivalent to Fig. 2c (see Supplementary Fig. 6). This effect results from the mid-infrared biasing alone, and therefore, it subtracts automatically from the differential transmission changes discussed in our work (see Methods for a detailed discussion). In any case, our subcycle and ultrabroadband analysis of interband electro-absorption provides complementary information as compared to studies of high-harmonics emission such as e.g., refs.^1–5^.

In summary, we have shown that purely off-resonant optical biasing of a bulk semiconductor can result in an extreme state of matter with a strongly modified electronic structure originating from Wannier–Stark localization. Requiring external fields far above stationary breakdown conditions, this state can exist only over intervals short enough to prevent the buildup of significant interband tunneling, resulting in subsequent avalanche multiplication after the acceleration of generated free electrons, as well as the polar lattice motion to accumulate. It will be fascinating to see whether future theoretical studies at an atomistic level and scattering experiments with few-femtosecond resolution can give further insight into the intricate relationship between the electronic structure of a solid-state crystal and its constituent atomic species that is indicated by our measurements and qualitative reasoning. In this context, the idea to extend our experiment to systems characterized by 2D or even 1D lattices will unveil the topological differences in wavefunction localization and formation of a ladder in optical transitions. Definitely, the transient Wannier–Stark state might provide attractive physical and even novel chemical functionalities owing to the extreme properties of its electronic system. For example, we had to work with extremely low near-infrared and visible pulse energies of 50 pJ in order to ensure that the transient transmission through the biased samples remained linear in the probe spectral intensity (see Methods). Spectacular non-linear optical phenomena occur already when the probe pulse energy is increased to a still minor value of 1 nJ. They are animated in Supplementary Movie 1 showing the relative changes in the probe spectra upon transmission through an 800-nm thin film of GaAs, which is scanned through the confocal region of the MIR bias field.

## Methods

### Experimental setup and calibration of mid-infrared bias fields

The phase-locked high-field biasing transients are provided by a multi-terahertz laser source based on a regenerative Ti:sapphire amplifier, parametric frequency conversion and difference frequency mixing in GaSe^24,25^. Near-infrared and visible probe pulses of a duration of 7 fs are generated by non-collinear parametric amplification^26^. The complete experimental setup is presented in Supplementary Fig. 1. Characterization of the few-cycle mid-infrared transients is carried out and checked in the following way: first, we determine the electric field amplitude as a function of time with ultrabroadband electro-optic sampling^27^ using a 30-µm-thick GaSe crystal as a detector and an 8-fs probe pulse centered at a wavelength of 1.2 μm derived from the ultrabroadband Er:fiber front-end. We then check the quantitative values for the electric field amplitude by measuring the mid-infrared average power and confocal cross section with a calibrated thermopile detector and razor blade. Taking into account the repetition rate of the system of 1 kHz and the intensity envelope from the square of the electric field trace, we obtain an alternative measure for maximum power and field amplitude. The values given for the electric field throughout this paper refer to the interior of the GaAs sample. They are obtained via the Fresnel transmission coefficient for the mid-infrared field at the air–GaAs interface.

Probe pulses have been focussed to a paraxial spot radius of 10 μm. We have checked carefully that our probe pulse energies of 50 pJ are low enough to warrant a linear response of the excited sample. Identical differential transmission changes are obtained up to probe pulse energies of 200 pJ. It is interesting to see what happens at yet higher probe pulse energies of 1 nJ. Here, strong non-linear phase shifts due to self-phase modulation of the probe occur, as discussed in Supplementary Movie 1.

The single-cycle biasing waveform in Fig. 2a has been attenuated to a peak electric field of 1.4 MV/cm by rotating the polarization of the signal OPA for MIR generation with a half-wave plate. The experimental spectra of differential transmission changes at 6.6 MV/cm and 10 MV/cm electric amplitude in Fig. 2b have been extracted from two local field extrema of the same MIR field waveform without attenuation. With these biasing pulses, we were able to precisely follow the contours at the field extrema, which display some curvature in the two-dimensional data sets due to residual chirp on the interband probe pulses (compare Fig. 2c). As a compromise, we achieved only a peak internal field of 10 MV/cm, corresponding to no attenuation, as compared to the 12 MV/cm few-cycle transient in Fig. 2c.

### GaAs sample structures and preparation

Single-crystal and intrinsic GaAs films of thickness 100 nm, 400 nm and 800 nm were grown on a <110>-oriented GaAs substrate by molecular beam epitaxy after deposition of a 50-nm-thick AlAs layer. Following a lift-off procedure^28^, GaAs films were van-der-Waals bonded to 250-μm-thick diamond substrates with negligible absorption from far-infrared to near-ultraviolet. We have carefully checked that MIR-induced differential transmission signals obtained on the bare diamond substrate were negligible as compared to the effects provided by the epitaxial GaAs layers.

### Theoretical modeling

Linear optical absorption spectra in the presence of strong THz or dc fields are obtained by numerical solutions of the semiconductor Bloch equations (SBE) including interband and intraband transitions^4,5,29,30^. For weak optical excitation, only the interband coherences, also called microscopic polarizations *p*_*k*_^*cv*^, between the valence and conduction bands need to be considered. Neglecting excitonic effects, SBE for the interband coherences read

3 $$\begin{array}{c} {\frac{\partial }{{\partial t}}p_k^{cv} = \frac{i}{\hbar }\left( {\varepsilon _k^c - \varepsilon _k^v} \right)p_k^{cv} + \frac{e}{\hbar }E_{{\mathrm{THz}}}\left( t \right)\nabla _kp_k^{cv} - \frac{i}{\hbar }E_{{\mathrm{opt}}}\left( t \right)d_k^{vc} - \frac{{p_k^{cv}}}{{T_2}}} \end{array}$$

where *ε*_*k*_^*c/v*^ are the energy dispersions of electronic states in the conduction (*c*) and valence (*v*) bands, respectively, *d*_*k*_^*vc*^ is the interband dipole matrix element and *T*_2_ is the dephasing time. We distinguish between the weak optical field *E*_opt_(*t*), which is treated as an ultrashort *δ*(*t*)-like pulse for probing interband transitions, and *E*_THz_(*t*), which is either the intense transient THz field or when chosen as constant in time, that is *E*_THz_(*t*) = *E*, models a dc field. Since the frequency of the THz field is much smaller than the frequency corresponding to resonant interband transitions, only the intraband acceleration resulting from *E*_THz_(*t*) is considered in Eq. (3). This intraband acceleration term proportional to $\nabla _k$ can be treated exactly by introducing a time-dependent wave vector^29,30^ i.e., *k* changes according to the acceleration theorem with a rate proportional to *E*_THz_(*t*):

4 $$\begin{array}{c} {k\left( t \right) = k\left( {t = - \infty } \right) - \frac{e}{\hbar }\mathop {\int }\limits_{ - \infty }^t E_{{\mathrm{THz}}}\left( {t^\prime } \right)\mathrm{d}t^\prime ,} \end{array}$$

The total optical polarization is given by the sum over all microscopic polarizations:

5 $$\begin{array}{c} {P\left( t \right) = \mathop {\sum }\limits_{k,c,v} \left( {d_k^{vc}} \right)^ \ast p_k^{cv}\left( t \right) + {\mathrm{c}}.{\mathrm{c}}.} \end{array}$$

In the models considered in this work, we include the lowest conduction band and either one or two valence bands. The dipole matrix elements are chosen as constants independent of *k* and band indices.

Since *E*_THz_(*t*) is linearly polarized, SBE are solved numerically by discretizing the electronic dispersion in this direction^4,5,31^. Fourier transforming the total polarization *P*(*t*), linear absorption is obtained as

6 $$\begin{array}{c} {\alpha _{1{\mathrm{D}}}\left( \omega \right) \propto \omega \;Im\left( {P\left( \omega \right)} \right).} \end{array}$$

The electronic band structure perpendicular to the direction of *E*_THz_(*t*) is described by effective mass dispersions. Such two-dimensional parabolic bands exhibit a constant density of states. Therefore, within this model, three-dimensional absorption changes of a bulk crystal are given by a frequency integral over the one-dimensional absorption corresponding to the direction of *E*_THz_(*t*), i.e.,

7 $$\begin{array}{c} {\alpha _{3{\mathrm{D}}}\left( \omega \right) \propto \mathop {\int }\limits_0^\omega \alpha _{1{\mathrm{D}}}\left( {\omega ^\prime } \right)d\omega ^\prime .} \end{array}$$

In the model outlined in Fig. 3, we describe the band structure of the lowest conduction and the heavy-hole band by tight-binding cosine dispersions with widths corresponding to the energy differences between Γ and L-points. For the more realistic model of Fig. 4, we use the generalized-gradient approximation to density-functional theory as implemented in Quantum Espresso^32^ to calculate the GaAs band structure. In this approach, the projector-augmented wave technique is exploited to model the electron-ion^33^, as well as the spin-orbit interaction^34^, and quasiparticle energy shifts are included at the level of a scissors operator^35^.

To verify that indeed the theoretical modeling evaluated for Figs 3, 4, in particular, the use of Eq. (3) is justified and very well captures the essential physics for the experimentally investigated conditions, several additional calculations have been performed. We, in particular, carried out simulations which include both intra- and interband excitations of the strong THz-pump field to infinite order and the weak optical probe field to first order. The complete set of SBE describing the strong-THz-pump weak-optical-probe experiments reads for our two-band model:

8 $$\begin{array}{c} {\frac{\partial }{{\partial t}}p_k^{cv} = \frac{i}{\hbar }\left( {\varepsilon _k^c - \varepsilon _k^v} \right)p_k^{cv} + \frac{e}{\hbar }E_{{\mathrm{THz}}}\left( t \right)\nabla _kp_k^{cv} - \frac{i}{\hbar }E_{{\mathrm{THz}}}\left( t \right)d_k^{vc}\left( {1 - 2n_k^c} \right) - \frac{{p_k^{cv}}}{{T_2}}} \end{array}$$

9 $$\begin{array}{c} {\frac{\partial }{{\partial t}}n_k^c = \frac{e}{\hbar }E_{{\mathrm{THz}}}\left( t \right)\nabla _kn_k^c + \frac{i}{\hbar }E_{{\mathrm{THz}}}\left( t \right)d_k^{vc}\left( {p_k^{cv} - \left( {p_k^{cv}} \right)^ \ast } \right)} \end{array}$$

10 $$\begin{array}{c} {\frac{\partial }{{\partial t}}\delta p_k^{cv} = \frac{i}{\hbar }\left( {\varepsilon _k^c - \varepsilon _k^v} \right)\delta p_k^{cv} + \frac{e}{\hbar }E_{{\mathrm{THz}}}\left( t \right)\nabla _k\delta p_k^{cv} + I_1 + I_2 + I_3 - \frac{{\delta p_k^{cv}}}{{T_2}}} \end{array}$$

11 $$\begin{array}{c} {\frac{\partial }{{\partial t}}\delta n_k^c = \frac{e}{\hbar }E_{{\mathrm{THz}}}\left( t \right)\nabla _k\delta n_k^c + \frac{e}{\hbar }E_{{\mathrm{opt}}}\left( t \right)\nabla _kn_k^c + \frac{i}{\hbar }E_{{\mathrm{opt}}}\left( t \right)d_k^{vc}\left( {p_k^{cv} - \left( {p_k^{cv}} \right)^ \ast } \right)} \\ { + \frac{i}{\hbar }E_{{\mathrm{THz}}}\left( t \right)d_k^{vc}\left( {\delta p_k^{cv} - \left( {\delta p_k^{cv}} \right)^ \ast } \right)} \end{array}$$

where the three inhomogeneities that appear in Eq. (10) are given by $I_1 = - \frac{i}{\hbar }E_{{\mathrm{opt}}}\left( t \right)d_k^{vc}\left( {1 - 2n_k^c} \right)$, $I_2 = \frac{i}{\hbar }E_{{\mathrm{THz}}}\left( t \right)d_k^{vc}2\delta n_k^c$ and $I_3 = \frac{e}{\hbar }E_{{\mathrm{opt}}}\left( t \right)\nabla _kp_k^{cv}\left( t \right).$ Equations (8) and (9) take both intra- and interband transitions originating from the THz field, *E*_THz_, to arbitrary order into account. Such sets of equations and extensions involving additional bands have been used in the recent literature to analyze THz-induced high-harmonic generation, as well as dynamic Bloch oscillations and the interference of Bloch electrons^4,5,31,36^. They describe also electron-hole-pair generation (proportional to $n_k^c$ and $\left( {1 - n_k^c} \right)$, respectively) induced by intense THz fields. The strong-THz-pump weak-optical-probe experiments require us to solve additional equations, which properly include the weak optical test pulse. Equations (10) and (11) include the optical probe pulse to first order, and the full set of the coupled Eqs. (8)–(11) needs to be evaluated to determine the THz-induced modifications of the optical properties as probed by the weak optical test pulse, *E*_opt_. The absorption viewed by the optical probe is determined by $\delta p_k^{cv}$.

The $\delta p_k^{cv}$ Eq. (10) includes three inhomogeneities *I*_1_, *I*_2_ and *I*_3_, which denote different physical effects: *I*_1_ is proportional to $\left( {1 - 2n_k^c} \right)$ and describes Pauli blocking, resulting in bleaching of the optical absorption arising from electronic occupations $n_k^c$ generated by the THz field, *I*_2_ is proportional to $2\delta n_k^c$ and describes the (optical/dynamical) Stark shift originating from the THz driving and *I*_3_ being proportional to $\nabla _kp_k^{cv}$ represents an unusual source term, where the optical field leads to an intraband acceleration.

In all our simulations, *I*_3 _turned out to be quite small, but was still included for completeness. Regarding the terms *I*_1_ and *I*_2_, we find that they modify the results only for very high THz field amplitudes and furthermore, for the experimental situations studied in this work, that *I*_1_ is significantly larger than *I*_2_. Exemplary results confirming these findings are shown in Supplementary Fig. 3. In total, our numerical simulations clearly demonstrate that for THz field amplitudes of at least 10 MV/cm, the negative region in the differential transmission is not significantly reduced by higher-order and interband transitions caused by an intense THz field and thus can be taken as an experimental indication for the onset of (transient) Wannier–Stark localization. It is furthermore important to note that our two-band model represents a worst-case scenario in this respect. In the experiment, the electron and hole occupations generated by an intense THz field will not stay in the lowest conduction and the highest valence bands. Instead, they will be further excited to the energetically higher conduction and the lower valence bands, respectively, which will actually reduce their contribution to the bleaching of the optical absorption in the frequency range that is investigated here. Thus the results obtained within the two-band model are expected to provide a lower estimate for the THz amplitude up to which Eq. (3) describes the relevant physical effects well. This is, however, already sufficient to justify the interpretation of the experimental results and the main finding, i.e., the observation of transient Wannier–Stark localization.

As a strong THz field leads to a very rapid intraband acceleration through the entire Brillouin zone, we use in these simulations a *k*-independent interband dipole matrix element of $d_k^{vc} = 2.25$ eÅ, which is the average of 3 eÅ, the typical value often used for near-bandgap excitation of GaAs, and 1.5 eÅ, which is obtained for transitions at the upper band edge considering the $\left( {\varepsilon _k^c - \varepsilon _k^v} \right)^{ - 1}$ scaling of the interband dipole matrix element. Therefore, this value is adequate and even slightly larger than the shift distance of GaAs, which amounts to approximately 2 Å at the fundamental bandgap^37,38^.

### Role of interband tunneling and high optical harmonics generation

Based on the theoretical arguments, differential transmission changes due to free electrons and holes generated via off-resonant interband excitation in the high THz bias are of minor importance up to peak fields in the order of 10 MV/cm. By measuring the differential changes in spectral transmission over an extended interval of time delays (see Supplementary Fig. 4), we can demonstrate that this statement holds even more in the experiment where higher valence and conduction bands are present instead of the two-band case considered theoretically.

In Supplementary Fig. 4, the non-resonant differential transmission change discussed in the main part of the paper shows up at the very bottom, around *t* = 0. While the details are not fully resolved due to the extended time interval, the abrupt change from bleaching (red) to induced absorption (blue) at *E*_pr_ = 2.1 eV remains clearly discernible. Very interestingly, hardly any transmission change is detectable for approximately 500 fs after the intense biasing transient has ended. It is only after this time interval that a massive bleaching (red) appears in almost the entire first interband transition region, indicating impressively that we are dealing here with two different physical effects. Our interpretation of the monotonous bleaching after *t* = 500 fs (note the absence of any induced absorption in this regime) is as follows: During the intense mid-infrared biasing around *t* = 0, a moderate density of electron-hole pairs is generated via interband tunneling. In contrast to the two-band theory where the carriers cannot escape in k-space, the high field amplitude leads to a giant gain in energy and eventually a very dilute electronic occupation over the entire band structure. Subsequently, those extremely hot carriers are losing energy via phonon emission and especially via impact ionization of additional electron-hole pairs over the fundamental gap. Together, carrier cooling and multiplication result in the arrival of broadband bleaching observed in Supplementary Fig. 4 later than *t* = 500 fs. In any case, the data clearly show that the initial transmission changes under direct mid-infrared bias are hardly influenced by those free carriers, and that they exhibit a rather different spectral signature.

Additional support for our claim of minor contributions to the signals during mid-infrared biasing due to generation of real carriers comes from Supplementary Fig. 5, where differential transmission changes, Δ*T*/*T*, measured at two different probe photon energies are plotted as a function of time. The data at *E*_pr_ = 1.9 eV (red line) show an oscillation with twice the mid-infrared driving frequency. The modulation depth of these temporal oscillations is close to 100 %, especially when taking into account also the finite duration of the probe pulse of 7 fs, as it is expected for a purely non-resonant and therefore quasi-instantaneous effect, which to first order is quadratic in the field. Instead, a gradual buildup of a positive background would indicate a significant influence of bleaching due to real carriers induced by the high bias. At a probe photon energy of 2.3 eV (blue line in Supplementary Fig. 5), a slight resonant background is indeed building up gradually, but it is of negative sign in Δ*T*/*T*. We attribute this signature to the transient broadening of the *E*_1_ and *E*_1_ + *Δ*_1_ gaps around 3 eV (see Fig. 2c) due to the presence of Coulomb scattering with the extremely hot carriers that have been induced. This interpretation also explains the clear decrease in the negative background seen already between *t* = 100 fs and 250 fs in Supplementary Fig. 5; the broadening of the zone-edge gap is reduced due to the buildup of screening and cooling of the carrier distribution which results in the reduction of phase space for carrier–carrier interaction.

We also want to address the negligible role of high-harmonics generation^1–5^ for the interpretation of our data. Under our biasing conditions, we can clearly see this effect also for the bulk semiconductor GaAs under study (see Supplementary Fig. 6). Nevertheless, we want to underline that this emission originates from the mid-infrared bias alone and it does not necessitate the presence of even a weak interband probe pulse. Since we are analyzing differential transmission spectra, the high-harmonics emission is subtracted from the data already in first order. Together with the fact that the absolute high-harmonics intensity is several orders of magnitude smaller than the intensity change corresponding to the differential transmission effects reported in Fig. 2, we can safely exclude any influence of this emission in those measurements.

### Data availability

The data that support the findings of this study are available from the corresponding author upon reasonable request.

## Electronic supplementary material

Supplementary Information

Description of Additional Supplementary Files

Supplementary Movie 1
