# Supplementary material for: Signatures of transient Wannier-Stark localization in bulk gallium arsenide
Source: Nat Commun. 2018 Jul 23;9:2890. doi: 10.1038/s41467-018-05229-x (PMC6056559; doi:10.1038/s41467-018-05229-x)
Supplement: Supplementary file 1 — Supplementary Information [file 41467_2018_5229_MOESM1_ESM.pdf]

Signatures of Transient Wannier-Stark Localisation in Bulk Gallium Arsenide

C. Schmidt et al.

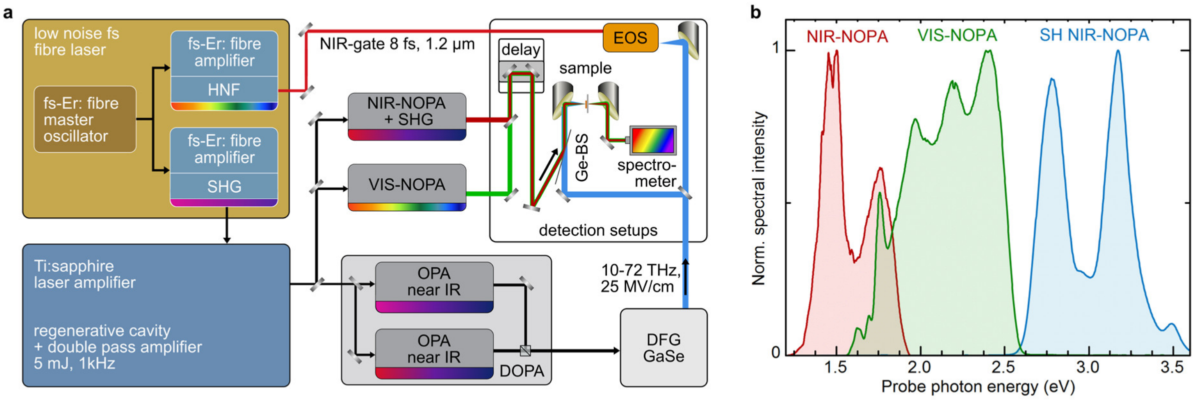

Supplementary Figure 1 *Experimental setup and interband probe spectra*. **a** A mode-locked Er:fibre master oscillator seeds two parallel branches equipped with femtosecond Er:fibre amplifiers. The top branch is equipped with a highly nonlinear fibre (HNF) and provides a near-infrared (NIR) gate pulse for electro-optic sampling (EOS) of the MIR phase-stable wave form. The lower branch is frequency doubled to a wavelength of 780 nm and seeds a regenerative Ti:sapphire amplifier with subsequent double-pass booster which generates 130-fs pulses of 5 mJ energy at a repetition rate of 1 kHz. A large part of this output is used to pump two parallel optical parametric amplifier (OPA) stages which are arranged in a compact double setup (DOPA) to provide tunable near-infrared pulses with minimum timing jitter. After combination to a single beam, difference frequency generation (DFG) in a GaSe nonlinear-optical crystal is used for generation of the phase-stable MIR high-field transients. They are focussed onto the sample structures with off-axis parabolic mirrors of focal length f = 15 mm and effective NA = 0.2. The remaining part of the Ti:sapphire amplifier output is exploited to pump a noncollinear optical parametric amplifier with visible emission (VIS-NOPA) and a setup which is optimized for the near infrared (NIR-NOPA). This branch is equipped with an optional stage for second harmonic generation (SHG). After an optical delay which sets the timing between MIR field transients and NIR/VIS probing, both pulse trains are superimposed by a germanium beam splitter (Ge-BS). The NIR/VIS pulses are then focussed into a spectrometer coupled to a CCD camera for spectrally-resolved detection of the probe photons. The MIR biasing transient is modulated by a mechanical chopper blade operating at a frequency of 125 Hz (not shown) which is locked to the 1-kHz pulse train and triggers readout of the CCD camera. In this way, we can obtain relative differential transmission changes $\Delta T/T$ by subtracting two subsequent spectra taken from the CCD camera and normalizing them to the spectrum obtained with no MIR pump pulses present. **b** Normalized spectral intensity versus photon energy for the probe spectra generated with the NIR-OPA (red), the fundamental of the VIS-NOPA (green) and the second harmonic (SH) of the NIR-OPA (blue). Extremely broadband SH generation was achieved with a BBO crystal as thin as 10 µm under a phase-matching angle of 29°.

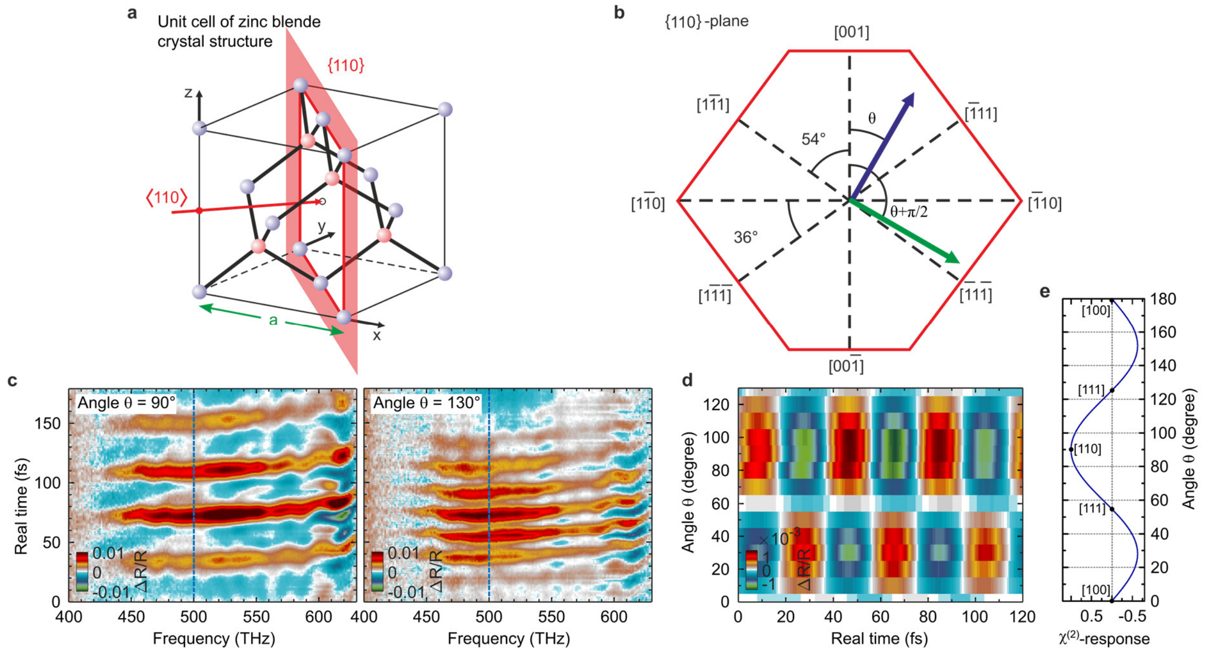

Supplementary Figure 2 *Gallium Arsenide lattice properties and electro-optic effects.* **a** The unit cell of the zincblende-type lattice structure of GaAs is shown in this plot with blue and red spheres representing the two different types of constituent atoms, respectively, and primary chemical bonds sketched by solid black lines. Thin black lines define the cubic shape of the unit cell with an edge length given by the lattice constant of *a* = 5.6532 Å. Our samples are non-standard because they were epitaxially grown in the <110> crystallographic direction (red arrow). When the MIR bias field hits the sample under normal incidence, the electric field vector lies in the {110} crystallographic plane (indicated by the red frame) and therefore, we can transiently bias the electronic structure along both the <111> and <100> high-symmetry directions by adequate rotation of the sample about its surface normal. **b** A cut through the first Brillouin zone along the {110} plane is depicted by the red lines. The angle between the electric bias field direction (blue arrow) and the [001] axis is defined by θ. The polarization of our probe pulses (green arrow) is always set perpendicular to the pump field. In this geometry, we can avoid influences due to the second-order optical nonlinearity of the non-centrosymmetric crystal which yields transmission changes that are linear in the electric bias field *E*. Two limiting cases are shown in **c**. In the left panel, θ = 90° and the bias field points along the low-symmetry [-110] direction of the crystal. Relative reflectivity changes Δ*R*/*R* at the surface of a <110>-oriented i-GaAs wafer are colour coded as a function of time and probe frequency. In this case, the temporal periodicity follows the electric bias field, indicating that the leading order in the nonlinearity is due to the electro-optic effect. In contrast, when adjusting θ to be 130° (right panel), the bias field direction is close to the high-symmetry [-11-1] axis of the crystal. Here, the signatures in Δ*R*/*R* are modulated with the second harmonic frequency of the biasing field transient, as it should be for a purely third-order nonlinear response. In this geometry, we can study the electronic structure changes due to Franz-Keldysh effects and Wannier-Stark localisation which depend on the absolute value of the field without contamination due to the relatively trivial second-order response induced by charge displacement about the axis of the Ga-As dimers. **d** Reflectivity changes Δ*R*/*R* as a function of orientation angle θ in the time interval during a high-field biasing transient. One can see that the signatures oscillating with the fundamental frequency of the MIR transient are absent for angles θ around 0°, 60° and 130° where the bias field points either in [100]- or [111]-equivalent directions. Here, only signatures oscillating to first order with the second-harmonic of the bias fields remain. These are the effects relevant to this study. **e** Calculation of the effective second-order response of the sample as a function of rotation angle θ, confirming that by symmetry, there is no contribution from $\chi^{(2)}$ when the bias field points into either the [111] or [100] crystallographic directions. Among those two possibilities, the [111] biasing direction is more suited for Wannier-Stark localisation because of a maximum lattice periodicity *D* and minimum width Δ of electronic energy bands (see Fig. 1b). Note that permanent damage occurs at the sample due to our MIR transient wave forms at a peak field of 15 MV/cm.

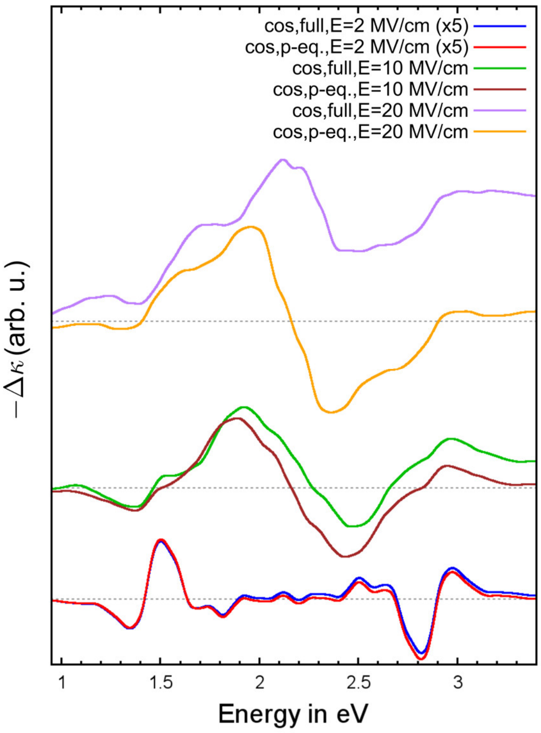

Supplementary Figure 3 *Including THz interband excitations to infinite order.* Negative extinction change -Δκ with respect to the unbiased case versus photon energy and for various strengths of the THz field calculated for the cosine-like two-band model used in Fig. 3. For a peak amplitude of the THz field of 2 MV/cm the differences between the approximate, Eq. (3), and the full treatment are hardly discernible. For 10 MV/cm, due to the bleaching of the optical absorption by THz-generated electron and hole occupations, some quantitative differences between the prediction of Eq. (3) and the full treatment occur. However, the negative minimum indicating the onset of Wannier-Stark localisation is still present when THz interband excitations are included. For an even larger THz field amplitude of 20 MV/cm, the THz-induced electron and hole occupations change the results qualitatively and, e.g., may disguise the optical probe of Wannier-Stark localisation, as the bleaching of the optical absorption results in a purely negative absorption change. It should be noted that even in this regime Wannier-Stark localisation may still be present, however, its unambiguous identification surely requires a more complex analysis. Note that in our experiments the GaAs sample suffers permanent damage starting at a peak field of 15 MV/cm. Also, in the Methods section related to Supplementary Figures 5 and 6 we investigate the influence of interband-generation of free carriers from an experimental point of view and argue that the influence is even less pronounced than in the results of the two-band model discussed here.

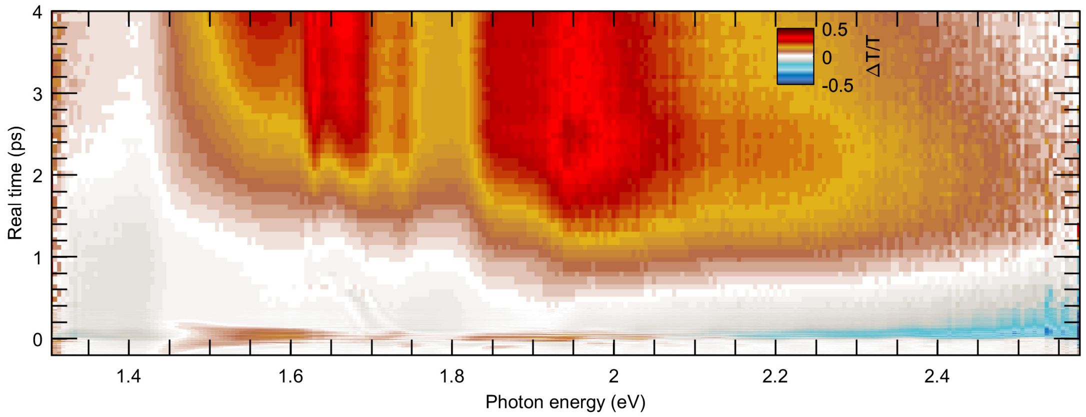

Supplementary Figure 4 *Signatures of free carriers from interband tunnelling.* Differential transmission changes ΔT/T color-coded as a function of probe photon energy and for an extended interval of pump-probe time delays up to 4 ps.

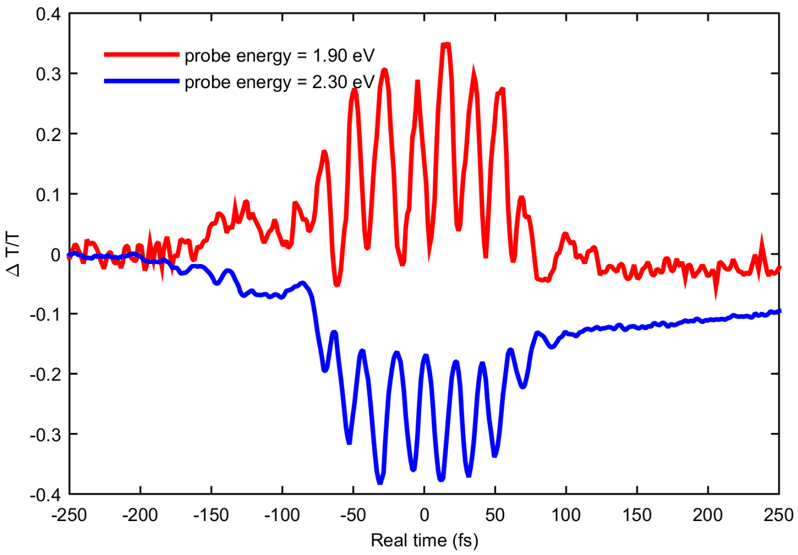

Supplementary Figure 5 *Dominance of adiabatic response during high-field biasing.* Differential transmission changes ΔT/T measured at probe photon energies of 1.9 eV (red line) and 2.3 eV (blue).

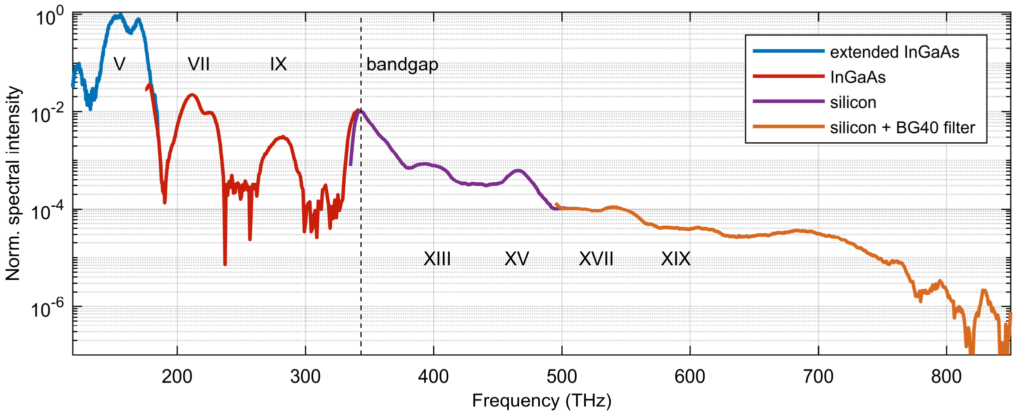

Supplementary Figure 6 *High-harmonics emission intensity* due to off-resonant mid-infrared biasing of GaAs under conditions identical to Fig. 2c. Second-order nonlinear effects are absent when pumping with a field oriented in (111)-direction. Therefore, only odd harmonics are visible but clear maxima are resolved up to 19th order. The last peak is followed by a broad continuum around a frequency of 700 THz due to the few-cycle character of the bias field. This feature is analogous to the generation of isolated attosecond pulses in gas targets pumped with near-infrared radiation.
