# Supplementary material for: Signatures of transient Wannier-Stark localization in bulk gallium arsenide
Source: Nat Commun. 2018 Jul 23;9:2890. doi: 10.1038/s41467-018-05229-x (PMC6056559; doi:10.1038/s41467-018-05229-x)
Supplement: Supplementary file 2 — Description of Additional Supplementary Files [file 41467_2018_5229_MOESM2_ESM.pdf]

Description of Additional Supplementary Files

File Name: Supplementary Movie 1
Description: *Giant optical nonlinearity in the Wannier-Stark regime*. This animation shows how the normalized differential transmission evolves as a function of time with respect to the MIR bias field and probe frequency when the sample is scanned through the confocal region of the setup. Here, a probe pulse energy of 1 nJ was used which is increased by a factor of 20 as compared to the measurements discussed in the main text. Both the distance of the GaAs sample from the confocal plane and the MIR peak field measured at this position are indicated in the black frame above the two-dimensional data sets, respectively. Far outside the focus, all recorded features are identical to the signatures found in Fig. 2a and also the sharp new absorption edge at 2.1 eV (500 THz) shows up in the interval closer than 0.2 mm to the focus where the peak electric fields are above 6 MV/cm. An additional effect arises in the region within 0.1 mm to the confocal plane where the MIR field exceeds 9 MV/cm: the measured values for $\Delta T/T$ are no longer independent of probe pulse energy as it should be in the linear limit of a differential transmission measurement. Instead, a completely new spectral pattern shows up which is still strongly modulated in sync with the extremal positions of the biasing field (i.e. along the vertical direction, compare MIR transient depicted at right). An intensity increase is found at frequency positions where the probe spectrum is minimal and a decrease where there are maxima, as evident from a comparison with the probe spectral intensity depicted in the bottom of the figure. This scenario is consistent with spectral redistribution of intensity due to self-phase modulation of the probe pulses. The fact that a considerable nonlinear phase shift is induced by 1 nJ pulses focussed to a spot radius of 10 µm onto a film of material as thin as 800 nm is consistent with a giant nonlinear refractive index of GaAs in the Wannier-Stark state. In the future, such extreme materials properties which can be induced only during the subcycle biasing intervals of intense optical driving fields might even find technical applications.
